# Incidence of Retinal Artery Occlusion and Related Mortality in Korea, 2005 to 2018

**DOI:** 10.1001/jamanetworkopen.2023.3068

**Published:** 2023-03-10

**Authors:** Daniel Duck-Jin Hwang, Kyung-Eun Lee, Yuwon Kim, Myoung-Suk Kim, Tyler Hyungtaek Rim, Mina Kim, Hasung Kim, Dae-Sung Kyoung, Ji In Park

**Affiliations:** 1 Department of Ophthalmology, Hangil Eye Hospital, Incheon, Korea; 2 Department of Ophthalmology, Catholic Kwandong University College of Medicine, Incheon, Korea; 3 Data Science Team, Evidnet Co Ltd, Seoul, Korea; 4 Singapore Eye Research Institute, Singapore National Eye Centre, Singapore; 5 Data Science Team, Hanmi Pharm Co Ltd, Seoul, Korea; 6 Department of Medicine, Kangwon National University Hospital, Kangwon National University School of Medicine, Chuncheon, Gangwon-do, Korea

## Abstract

**Importance:**

It remains unclear whether comorbidities in patients with retinal artery occlusion (RAO), a rare retinal vascular disorder, differ by subtype and whether mortality is higher.

**Objective:**

To examine the nationwide incidence of clinically diagnosed, nonarteritic RAO, causes of death, and mortality rate in patients with RAO compared with that in the general population in Korea.

**Design, Setting, and Participants:**

This retrospective, population-based cohort study examined National Health Insurance Service claims data from 2002 to 2018. The population of South Korea was 49 705 663, according to the 2015 census. Data were analyzed from February 9, 2021, to July 30, 2022.

**Main Outcomes and Measures:**

The nationwide incidence of any RAO, including central RAO (CRAO; *International Statistical Classification of Diseases and Related Health Problems, Tenth Revision [ICD-10]* code, H34.1) and noncentral RAO (other RAO; *ICD-10* code, H34.2) was estimated using National Health Insurance Service claims data from 2002 to 2018, with 2002 to 2004 as the washout period. Furthermore, the causes of death were evaluated and the standardized mortality ratio was estimated. The primary outcomes were the incidence of RAO per 100 000 person-years and the standardized mortality ratio (SMR).

**Results:**

A total of 51 326 patients with RAO were identified (28 857 [56.2%] men; mean [SD] age at index date: 63.6 [14.1] years). The nationwide incidence of any RAO was 7.38 (95% CI, 7.32-7.44) per 100 000 person-years. The incidence rate of noncentral RAO was 5.12 (95% CI, 5.07-5.18), more than twice that of CRAO (2.25 [95% CI, 2.22-2.29]). Mortality was higher in patients with any RAO than in the general population (SMR, 7.33 [95% CI, 7.15-7.50]). The SMR for CRAO (9.95 [95% CI, 9.61-10.29]) and for noncentral RAO (5.97 [95% CI, 5.78-6.16]) showed a tendency toward a gradual decrease with increasing age. The top 3 causes of death in patients with RAO were diseases of the circulatory system (28.8%), neoplasms (25.1%), and diseases of the respiratory system (10.2%).

**Conclusions and Relevance:**

This cohort study found that the incidence rate of noncentral RAO was higher than that of CRAO, whereas SMR was higher for CRAO than noncentral RAO. Patients with RAO show higher mortality than the general population, with circulatory system disease as the leading cause of death. These findings suggest that it is necessary to investigate the risk of cardiovascular or cerebrovascular disease in patients newly diagnosed with RAO.

## Key Points

**Question:**

What is the nationwide incidence of clinically diagnosed retinal artery occlusion, and corresponding mortality and cause of death, compared with that in the general population in Korea?

**Findings:**

Based on claims data from 2002 to 2018 in Korea, this cohort study found that the incidence of retinal artery occlusion was 7.38 per 100 000 person-years, and that mortality was significantly higher in patients with retinal artery occlusion (standardized mortality ratio, 7.33), most commonly due to cardiovascular or cerebrovascular disease.

**Meaning:**

These findings suggest that the risk of cerebrovascular or cardiovascular disease in patients with newly diagnosed retinal artery occlusion must be investigated.

## Teaser

This cohort study examines the nationwide incidence of clinically diagnosed, nonarteritic retinal artery occlusion as well as the causes of death and mortality rate in patients with retinal artery occlusion compared with those in the general population in Korea.

## Introduction

Retinal artery occlusion (RAO) is a rare retinal vascular disorder that can cause severe visual impairment.^1,2,3^ RAO can be divided into central retinal artery occlusion (CRAO) and other RAO (ie, noncentral RAO), depending on the type of retinal vessel affected.^2,4,5^ The most common cause of RAO is thromboembolism from a large artery or the heart,^2,4^ and accumulated evidence shows a strong association between RAO and stroke and cardiovascular diseases (eg, myocardial infarction) and valvular heart disease.^4,6,7,8^ However, it is unclear whether there is a difference in comorbidities, including cardiovascular diseases, according to RAO subtype, and whether there is a difference in mortality compared with that in the general population.^9,10,11,12^ Furthermore, there are few reports on CRAO incidence in large population groups^3,13,14,15^; in particular, data on noncentral RAO is scarce, with only one report on annual incidence.^5^ Additionally, there are conflicting reports regarding whether CRAO or noncentral RAO is more prevalent.^5,16^

South Korea's National Health Insurance System (NHIS) enables large-scale epidemiological studies using a medical claims database of the entire Korean population.^14,17,18^ Research using NHIS data, as a nationwide cohort study across the entire population of all ages, is less likely to have a selection bias than studies based on data from hospitals or specific regions. Given the large sample size and reliable diagnoses, unbiased estimations drawn from this nation-level data may provide useful information on the epidemiologic features of patients with RAO. Therefore, this study aimed to investigate the incidences of clinically diagnosed CRAO and noncentral RAO using NHIS data, as well as the comorbidities, all-cause mortality, and causes of death in patients with RAO.

## Methods

### Study Design and Data Sources

This retrospective cohort study was designed on March 7, 2020, and used data from the Korean NHIS database from January 1, 2002, to December 31, 2018. Additionally, the 2015 census^50^ was used to define the entire population of Korea as the population at risk, and the causes of death were investigated using Statistics Korea data. Details regarding the NHIS database,^17^ which covers all residents of Korea; Statistics Korea, and the definitions (diagnostic codes) of CRAO,^14,18^ noncentral RAO,^5^ and comorbidities^19^ are provided in the eMethods in Supplement 1.

The study was approved by the institutional review board of Kangwon National University Hospital. It adhered to the tenets of the Declaration of Helsinki^20^ and was conducted in accordance with the Strengthening the Reporting of Observational Studies in Epidemiology (STROBE) reporting guideline.^21^ The requirement for written consent was waived because of the retrospective nature of the study conducted using a preconstructed deidentified data set.

### Participants

To remove preexisting cases of RAO, we excluded cases with a diagnostic code for RAO during the first 3 years of the study period (washout period, 2002 to 2004)^22^; the remaining cases had a disease-free period of at least 3 to 16 years prior to the index event. Additionally, we excluded cases with the diagnostic code for giant cell arteritis (GCA; M31.6, n = 33) in their claims.^14^

### Statistical Analysis

The person-time incidence rates for 2005 to 2018 were calculated as the number of people who developed CRAO and noncentral RAO divided by the total person-time at risk during the study period. Therefore, in this analysis, person-years were counted after the incidence time. To estimate incidence rates, we used a direct standardization method for age and sex, with the 2015 census population in Korea used as the standard population.^18^ Using these estimated standardized incidence rates, we calculated the annual incidence rates of CRAO and noncentral RAO for 2005 to 2018 and estimated the 95% CIs based on the Poisson distribution. χ^2^ analysis was used to compare the observed incidence rates between CRAO and noncentral RAO.

Standardized mortality ratios (SMRs) with 95% CIs were calculated to compare the mortality of patients with RAO to that of the general Korean population. The SMR is a ratio of the deaths of patients with RAO vs the deaths in the general population as captured by Statistics Korea.

Statistical analyses were performed using SAS version 7.0 (SAS Institute). Two-sided *P* < .05 was considered significant. Data were analyzed from February 9, 2021, to July 30, 2022.

## Results

We identified 51 326 patients with incident RAO (28 857 [56.2%] men; mean [SD] age, 63.6 [14.1] years). Among these patients, 15 684 [30.6%] had CRAO (9220 [58.8%] men; mean [range] age, 63.9 [0-98] years) and 35 642 [69.4%] had noncentral RAO (19 637 [55.1%] men; mean [range] age, 63.4 [0-102] years) (Table 1). Hypertension, cardiac valvular disorders, ischemic stroke, and chronic kidney disease were significantly more common in the CRAO group, whereas ischemic heart disease and dyslipidemia were significantly more common in the noncentral RAO group. Antithrombotic agent, antiplatelet drug, anticoagulant, and fibrinolytic agent use was significantly more common in the CRAO group than in the noncentral RAO group.

**Table 1.** Baseline Characteristics of Patients With RAO

Characteristic	No. (%)			*P* value
	Total RAO (N = 51 326)	CRAO (n = 15 684)	Noncentral RAO (n = 35 642)	
Age, mean (SD), y	63.6 (14.1)	63.9 (14.9)	63.41 (13.7)	<.001
Sex				
Male	28 857 (56.2)	9220 (58.8)	19 637 (55.1)	<.001
Female	22 469 (43.8)	6464 (41.2)	16 005 (44.9)	
Comorbidity				
Hypertension	31 930 (62.2)	9872 (62.9)	22 058 (61.9)	.02
Dyslipidemia	29 674 (57.8)	8407 (53.6)	21 267 (59.7)	<.001
Diabetes	23 583 (45.9)	7121 (45.4)	16 462 (46.2)	.10
Ischemic heart disease	13 071 (25.5)	3881 (24.7)	9190 (25.8)	.01
Ischemic stroke	7162 (14.0)	2579 (16.4)	4583 (12.9)	<.001
Heart failure	4261 (8.3)	1356 (8.7)	2905 (8.2)	.06
Chronic kidney disease	2432 (4.7)	802 (5.1)	1630 (4.6)	.008
Arterial fibrillation and flutter	1941 (3.8)	613 (3.9)	1328 (3.7)	.32
Hemorrhagic stroke	671 (1.3)	223 (1.4)	448 (1.3)	.13
Cardiac valvular disorders	601 (1.2)	214 (1.4)	387 (1.1)	.007
Drug use				
Antiplatelet drugs	18 659 (36.4)	6107 (38.9)	12 552 (35.2)	<.001
Anticoagulants	3458 (6.7)	1188 (7.6)	2270 (6.4)	<.001
Fibrinolytic agents	128 (0.2)	64 (0.4)	64 (0.2)	<.001

Abbreviations: CRAO, central retinal artery occlusion; RAO, retinal artery occlusion.

**Figure 1.**
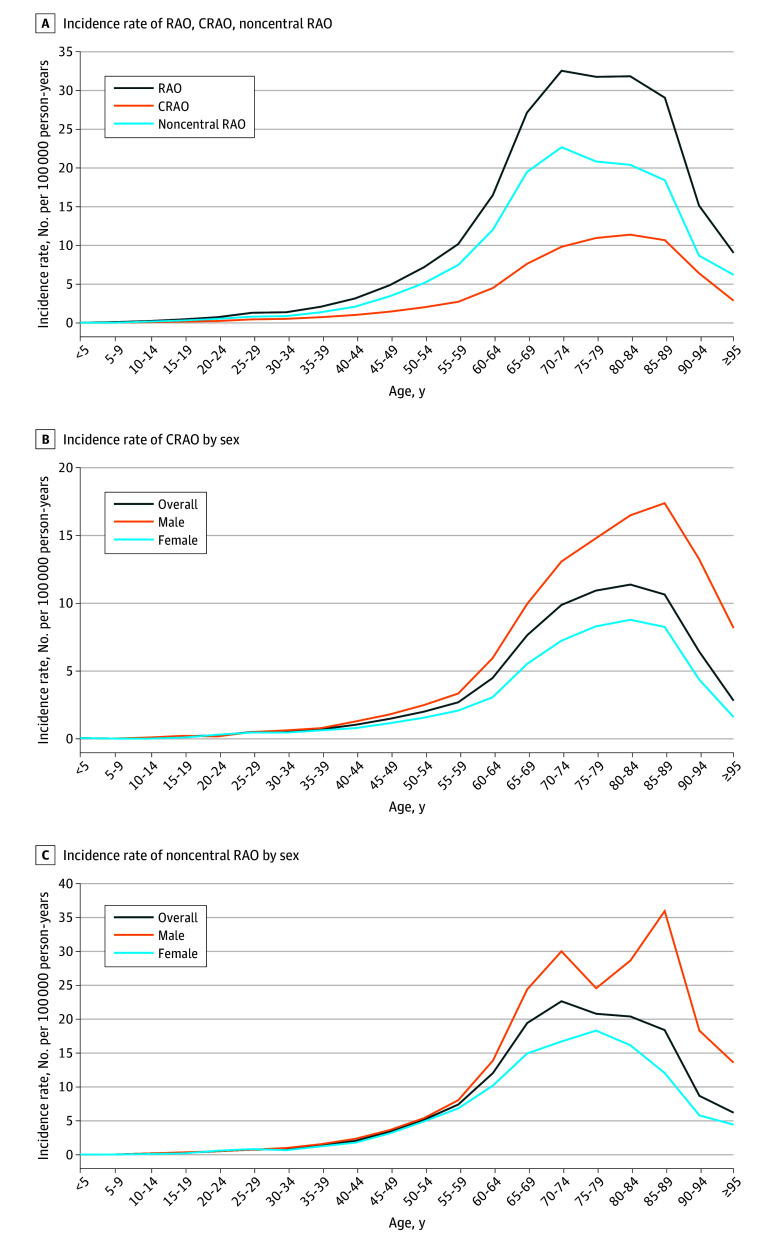
Incidence rates of RAO, CRAO, and Noncentral RAO A, Incidence rates of RAO, CRAO, and noncentral RAO in the Korean population from 2005 to 2018 (per 100 000 person-years). B, Incidence rate of CRAO by sex in the Korean population for the same period (per 100 000 person-years). C, Incidence rate of noncentral RAO by sex in the Korean population for the same period (per 100 000 person-years). CRAO indicates central retinal artery occlusion; RAO, retinal artery occlusion.

The mean incidence rate of any RAO was 7.38 (95% CI, 7.32-7.44), CRAO was 2.25 (95% CI, 2.22-2.29), and noncentral RAO was 5.12 (95% CI, 5.07-5.18) per 100 000 person-years (Table 2). The incidence rate ratio of CRAO-to-noncentral RAO was 0.44 (95% CI, 0.43-0.45). Incidence rates increased exponentially with age (Figure 1), and were highest at 70 to 74 years of age for any RAO, 80 to 84 years for CRAO, and 70 to 74 years for noncentral RAO.

**Table 2.** Frequencies and Incidence Rates of RAO Among Residents of Korea, 2005 to 2018

Age category, y	General population^a^	Incidence^b^						CRAO-to–noncentral RAO, IRR (95% CI)
	Total	RAO		CRAO		Noncentral RAO		
		No.	Incidence (95% CI)	No.	Incidence (95% CI)	No.	Incidence (95% CI)	
<5	223 5397	21	0.07 (0.04-0.10)	16	0.05 (0.03-0.08)	5	0.02 (0.01-0.04)	2.50 (0.90-6.97)
5-9	2 252 950	22	0.07 (0.04-0.11)	7	0.02 (0.01-0.05)	15	0.05 (0.03-0.08)	0.40 (0.16-1.00)
10-14	2 418 360	84	0.25 (0.20-0.31)	24	0.07 (0.05-0.11)	60	0.18 (0.14-0.23)	0.39 (0.24-0.63)
15-19	3 170 545	218	0.49 (0.43-0.56)	75	0.17 (0.13-0.21)	143	0.32 (0.27-0.38)	0.53 (0.40-0.71)
20-24	3 385 936	366	0.77 (0.70-0.86)	116	0.25 (0.20-0.29)	250	0.53 (0.46-0.60)	0.47 (0.38-0.59)
25-29	3 027 896	546	1.29 (1.18-1.40)	206	0.49 (0.42-0.56)	340	0.80 (0.72-0.89)	0.61 (0.51-0.73)
30-34	3 611 034	710	1.41 (1.30-1.51)	277	0.55 (0.49-0.62)	433	0.86 (0.78-0.94)	0.64 (0.55-0.75)
35-39	3 783 589	1122	2.12 (2.00-2.25)	374	0.71 (0.64-0.78)	748	1.41 (1.31-1.52)	0.50 (0.44-0.57)
40-44	4 215 921	1843	3.12 (2.98-3.27)	618	1.05 (0.97-1.13)	1225	2.08 (1.96-2.20)	0.50 (0.46-0.56)
45-49	4 266 941	2914	4.88 (4.70-5.06)	881	1.48 (1.38-1.58)	2033	3.40 (3.26-3.56)	0.44 (0.40-0.47)
50-54	4 145 976	4159	7.17 (6.95-7.39)	1171	2.02 (1.90-2.14)	2988	5.15 (4.97-5.34)	0.39 (0.37-0.42)
55-59	386 3095	5483	10.14 (9.88-10.42)	1457	2.69 (2.56-2.84)	4026	7.45 (7.22-7.68)	0.36 (0.34-0.38)
60-64	2 758 941	6368	16.50 (16.10-16.91)	1724	4.47 (4.26-4.68)	4644	12.03 (11.69-12.38)	0.37 (0.35-0.39)
65-69	2 117 875	8033	27.13 (26.54-27.73)	2269	7.66 (7.35-7.98)	5764	19.46 (18.96-19.97)	0.39 (0.38-0.41)
70-74	1 760 932	8004	32.52 (31.81-33.24)	2427	9.85 (9.46-10.25)	5577	22.65 (22.06-23.25)	0.44 (0.42-0.46)
75-79	1 356 014	6022	31.77 (30.97-32.58)	2073	10.93 (10.46-11.41)	3949	20.82 (20.18-21.48)	0.52 (0.50-0.55)
80-84	810 891	3603	31.79 (30.76-32.84)	1292	11.39 (10.78-12.03)	2311	20.38 (19.55-21.22)	0.56 (0.52-0.60)
85-89	371 527	1510	29.07 (27.62-30.57)	554	10.66 (9.79-11.58)	956	18.39 (17.25-19.60)	0.58 (0.52-0.64)
90-94	124 111	263	15.15 (13.37-17.09)	112	6.45 (5.31-7.76)	151	8.69 (7.36-10.20)	0.74 (0.58-0.95)
≥95	27 732	35	9.02 (6.28-12.54)	11	2.83 (1.42-5.07)	24	6.18 (3.96-9.20)	0.46 (0.22-0.95)
Total	49 705 663	51 326	7.38 (7.32-7.44)	15684	2.25 (2.22-2.29)	35642	5.12 (5.07-5.18)	0.44 (0.43-0.45)

Abbreviations: CRAO, central retinal artery occlusion; IRR, incidence rate ratio; RAO, retinal artery occlusion.

^a^ Information on the Korean population was based on 2015 census data from the Korean Statistical Information Service.

^b^ Incidence rate per 100 000 person-years.

Overall, the incidence rate of RAO was 1.29 times higher in men (8.31 [95% CI, 8.21-8.41] per 100 000 person-years) than in women (6.45 [95% CI, 6.37–6.54] per 100 000 person-years) (eTable 1 in Supplement 1). The incidence rate for RAO was highest at 80 to 84 and 75 to 79 years of age in men and women, respectively, and was higher in men than in women for most age groups. The male-to-female ratio ranged from 0.77 at 20 to 24 years of age to 3.61 at greater than 95 years of age, respectively. Overall, the incidence rate of CRAO was 1.43 times higher in men (2.65 [95% CI, 2.60-2.71] per 100 000 person-years) than in women (1.86 [95% CI, 1.81-1.90] per 100 000 person-years) (*P* < .001) (eTable 2 in Supplement 1). However, women had a higher CRAO incidence rate than men at 20 to 24 years of age (male-to-female ratio: 0.64, *P* = .02). The CRAO incidence rate was highest at 85 to 89 years of age in men and 80 to 84 years of age in women. The incidence rate of noncentral RAO was 1.23 times higher in men (5.65 [95% CI, 5.57-5.73] per 100 000 person-years) than in women (4.60 [95% CI, 4.52–4.67] per 100 000 person-years) (*P* < .001) (eTable 3 in Supplement 1). The RAO incidence rate was highest at 85 to 89 years of age in men and 75 to 79 years of age in women.

**Figure 2.**
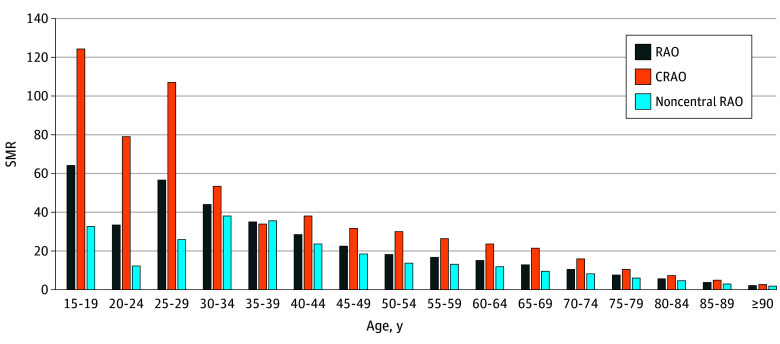
Standardized Mortality Ratios (SMRs) of RAO in Korea From 2005 to 2018 The SMR peaked in the teens and 20s, and gradually decreased thereafter for both men and women. The SMR of central retinal artery occlusion (CRAO) tended to be higher than that of noncentral RAO.

We identified 7107 deaths (4549 in men [64.0%]). The SMR was 7.33 (95% CI, 7.15-7.50) across all ages, and was higher in women (8.00 [95% CI, 7.69-8.31]) than in men (6.99 [95% CI 6.79-7.20]) (Figure 2; eTable 4 in Supplement 1). The SMR peaked in the teens and 20s, and gradually decreased thereafter for both men and women. Among patients with CRAO, we identified 3289 deaths (2134 in men [64.9%]). The SMR was 9.95 (95% CI, 9.61-10.29) for all ages and was higher in women (10.48 [95% CI, 9.87-11.08]) than in men (9.69 [95% CI, 9.28-10.10]) (eTable 5 in Supplement 1). Among patients with noncentral RAO, we identified 3818 deaths (2415 in men [63.3%]). The SMR was 5.97 (95% CI, 5.78-6.16) for all ages and was higher in women (6.69 [95% CI, 6.34-7.04]) than in men (5.61 [95% CI, 5.39-5.84]) (eTable 6 in Supplement 1).

**Figure 3.**
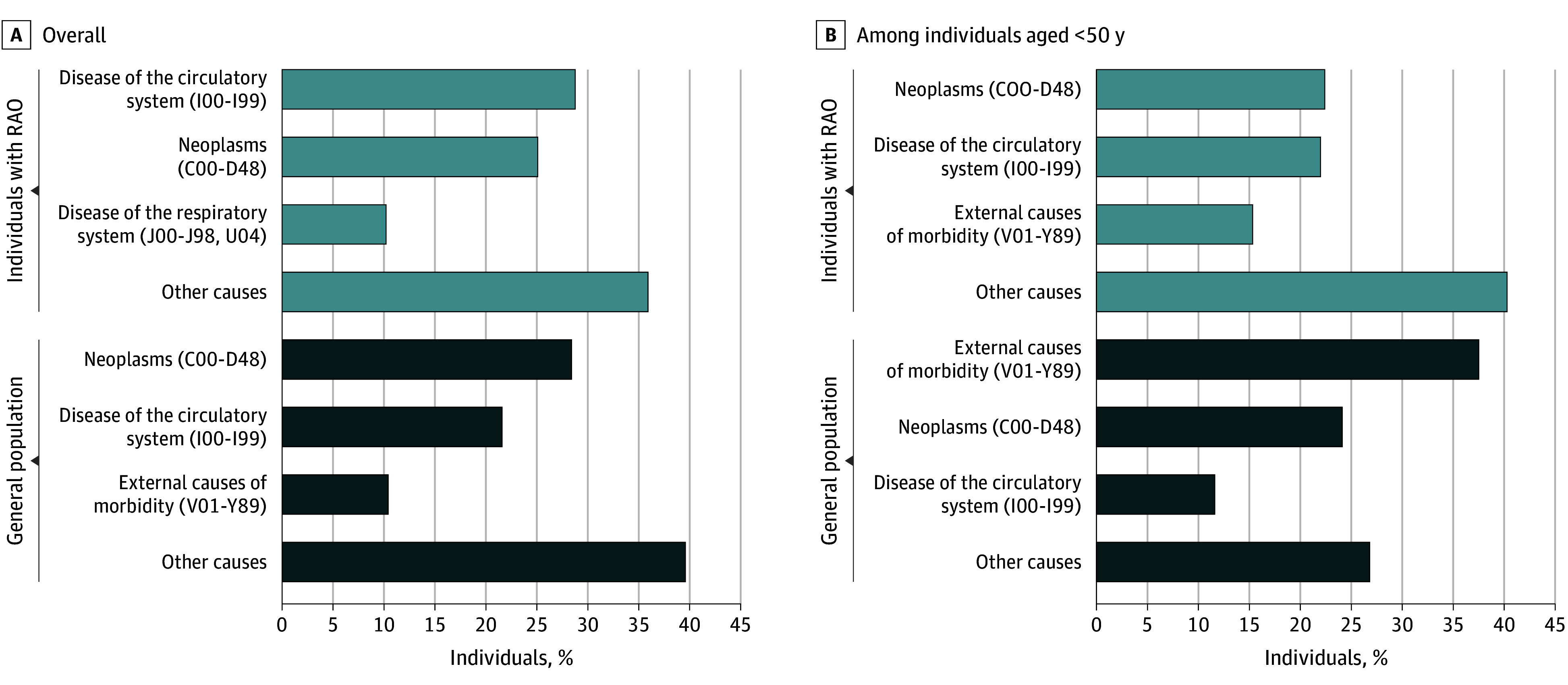
Top 3 Causes of Death Among Patients With Retinal Artery Occlusion (RAO) (2005-2018) and the General Population (2015) in Korea Among patients with RAO, diseases of the circulatory system were the most common cause of death, followed by neoplasms and diseases of the respiratory system. Among patients with RAO aged less than 50 years of age, neoplasms accounted for the largest proportion of deaths, followed by diseases of the circulatory system and external causes of morbidity. In contrast, in the general population, neoplasms were the most common cause of death, followed by diseases of the circulatory system and external causes of morbidity. In the general population aged less than 50 years, external causes of morbidity was the most common cause of death, followed by neoplasms and diseases of the circulatory system.

Among patients with RAO, diseases of the circulatory system (I00-I99; 28.8%) were the most common cause of death, followed by neoplasms (C00-D48; 25.1%) and diseases of the respiratory system (J00-J98, U04; 10.2%) (Figure 3; eTable 7 in Supplement 1). In contrast, in the general population, neoplasms (C00-D48; 28.4%) were the most common cause of death, followed by diseases of the circulatory system (I00-I99; 21.6%), and external causes of morbidity (V01-Y89; 10.4%). All causes of death in patients with RAO and specific diseases for the top 3 categories are listed in eTable 8 and eTable 9 in Supplement 1, respectively. Among diseases of the circulatory system, acute myocardial infarction (I21; 18.6%) had the highest frequency, followed by cerebral infarction (I63; 15.9%).

Of the 7053 deaths among patients with RAO, 258 (3.7%) occurred at younger than 50 years of age; the cause of death in this subgroup was additionally investigated (Figure 3; eTable 7 in Supplement 1). In this subgroup, neoplasms (C00-D48; 22.4%) accounted for the largest proportion of deaths, followed by diseases of the circulatory system (I00-I99; 22.0%) and external causes of morbidity (V01-Y89; 15.3%). In contrast, in the general population aged less than 50 years, external causes of morbidity (V01-Y89; 37.5%) was the most common cause of death, followed by neoplasms (C00-D48; 24.1%) and diseases of the circulatory system (I00-I99; 11.6%). All causes of death in patients with RAO aged less than 50 years are listed in eTable 10 in Supplement 1.

## Discussion

To our knowledge, this is the first nationwide study on the incidence, age at diagnosis, mortality, and cause of death in clinically diagnosed RAO, including epidemiologic characteristics across the entire population of all ages in Korea. The incidence of RAO from 2005 to 2018 was 7.38 per 100 000 person-years across all age groups, and the incidence of noncentral RAO was higher than that of CRAO. The incidence of both CRAO and noncentral RAO was highest at 70 to 80 years of age and was higher in men than in women. The SMR was 9.95 and 5.97 for CRAO and noncentral RAO, respectively, and the most common cause of death for both types was circulatory system disease.

The incidence of CRAO was 2.25 per 100 000 person-years in the present study and 1.80^14^ and 2.00^18^ per 100 000 person-years in 2 prior investigations using Korean population data. Despite differences in the length of the research period (14 years in the present study vs 4 years^14^ and 12 years^18^) and wash-out period (3 years in the present study vs 1 year^14^ and 2 years^18^), the incidences were similar. Furthermore, the incidence of CRAO in the present study was comparable with that in studies conducted in other countries,^3,5^ including Germany (n = 3 262 617; incidence, 2.7 per 100 000 person-years) and the United States (specifically, Minnesota; n = 106 470; incidence, 1.90 per 100 000 person-years), but was higher than that in a Croatian study^13^ (n = 465 947; incidence, 0.7 per 100 000 persons). The prevalence was highest at 80 to 84 years of age, similar to findings from studies conducted in the United States (75 to 84 years),^3^ Korea (80 to 84 years),^14,18^ and Germany (80 to 84 years),^5^ but higher than that in the Croatian study (60 to 69 years).^13^

In the present nationwide study, the incidence of noncentral RAO was 5.12 per 100 000 person-years. A hospital-based study,^16^ comprising 187 patients with RAO, reported that CRAO accounted for more than half of all RAO cases (57%); noncentral RAO, including cilioretinal artery occlusion, accounted for 43%. Few epidemiological reports have reported the incidence of noncentral RAO using claims data.^5^ A recent study conducted in Baden-Wuerttemberg, Germany (n = 4 104 201) reported an incidence of 4.5 per 100 000 person-years, which was higher than the incidence of CRAO in the same region at 2.7 per 100 000 person-years.^5^ In the present study, the incidence of noncentral RAO was also higher than that of CRAO, suggesting that the incidence of CRAO does not exceed more than half of total RAO incidence.

Previous studies on mortality in patients with RAO largely show inconsistent results due to relatively small sample sizes and short follow-up periods.^12,23,24,25,26^ Individual studies observed mortality in 29.6% of patients (n = 98; mean age, 64 years; mean follow-up, 4.2 years),^24^ 22.9% (n = 131; mean age, 70 years; follow-up, 11 years),^12^ and 5.4% (n = 221; mean age, 66.1; mean follow-up, 2.2 years).^25^ Additionally, a study that used pooled data from 2 population-based cohort studies (the Beaver Dam Eye Study [BDES] and Blue Mountains Eye Study [BMES]) with study periods of over 10 to 12 years, reported that the mortality of patients who developed RAO (56% [n = 111]; mean age: BDES, 69.0 years; BMES, 68.2 years) was almost twice that of patients without RAO.^23^ In contrast, a study by De Potter and Zografos^26^ (n = 151) did not show a statistically significant difference in mortality between patients with RAO and age- and sex-matched controls. In the present study, 7107 deaths were observed among 51 326 patients with newly diagnosed RAO during the 14-year study period, resulting in 13.8% mortality. Additionally, the SMR of patients with RAO was 7.33, indicating higher mortality compared with that in the general population. Furthermore, the SMR was higher for CRAO than for noncentral RAO, and higher in women than in men; however, the reason for the sex difference is unclear. Additionally, patients with RAO aged less than 50 years had a high SMR (>20), and patients aged greater than 50 years showed a tendency toward a gradual decrease in the SMR with increasing age.

Common causes of death in RAO reported in previous studies include coronary events (n = 17; 58.6%)^24^ and cardiovascular-related (n = 33; 29.7%) or stroke-related (n = 13; 11.7%) events,^23^ which is similar to the present study results. In the present study, cardiovascular or cerebrovascular mortality was the leading cause of death (28.8%); among these cases, acute myocardial infarction was the most frequent cause of death. In contrast, neoplasms (28.4%) and diseases of the circulatory system (21.6%) accounted for the 2 largest proportions of death in the general population, suggesting that mortality in patients with RAO is highly related to cardiovascular or cerebrovascular disease, as previously reported.^12,27^ Additionally, diseases of the circulatory system accounted for the second largest proportion (22%) of death in patients with RAO aged less than 50 years, but accounted for the third largest proportion (11.6%) in the general population aged less than 50 years. Furthermore, the high mortality rate (SMR >20) and large proportion of death due to cardiovascular or cerebrovascular disease (22%) in patients with RAO aged less than 50 years may reflect premature death in a high-risk group with diseases of circulatory system. This observation is consistent with the clinical association of RAO with cardiovascular and cerebrovascular disease, and it suggests the need to investigate the risk of developing cardiovascular and cerebrovascular disease in patients with newly diagnosed RAO.

Unlike a previous report on CRAO,^14^ comorbidity data were available for analysis in the present study. We found that diabetes, heart failure, arterial fibrillation and flutter, and hemorrhagic stroke had similar frequencies in CRAO and noncentral RAO. In contrast, hypertension, heart valve disease, chronic kidney disease, and ischemic stroke were more frequent in CRAO, while ischemic heart disease and dyslipidemia were more frequent in noncentral RAO. Part of the difference between CRAO and noncentral RAO might be related to the size of the emboli.^12,28^ Ischemic stroke may be associated with larger emboli while ischemic heart disease and small vessel disease may be associated with smaller emboli.^28,29^ In a retrospective, single-center study of 131 patients with CRAO or branch RAO (BRAO), triglyceride levels were higher in BRAO than in CRAO.^12^ Furthermore, cerebral small vessel disease (SVD) without large vessel disease is frequently present in nonembolic BRAO.^30^ Additionally, lesions of small vessels in the central nervous system are more common in patients with hypertriglyceridemia.^31^ These findings may suggest that SVD should be considered in the etiology of BRAO, although the reason for the higher prevalence of dyslipidemia in noncentral RAO than in CRAO is unclear.^12,30,31^

Over the past decade, numerous studies have highlighted the high risk of stroke and other cardiovascular diseases in patients with RAO.^6,7,8,9,32,33^ Most patients with RAO are diagnosed with major vascular risk factors and cardiovascular diseases, including hypertension, diabetes, dyslipidemia, ischemic heart disease, peripheral artery disease, atrial fibrillation, and heart failure.^6,7,8,9,32,33,34,35,36,37^ As in previous reports, our study also showed that these cardiovascular diseases were prevalent at the time of diagnosis of RAO. Retinal Vein Occlusion (RVO) also shares several risk factors with RAO, and there are some similarities in their pathophysiologies.^36,38^ It is generally accepted that RAO is caused by atherosclerosis, mainly emboli originating from plaques, whereas the primary pathophysiology associated with RVO was changes in the pressure gradients in the eyes.^38^ Ørskov et al^38^ showed that RAO was more strongly correlated with arterial hypertension, heart failure, ischemic heart disease, peripheral artery disease, and stroke than RVO in their case-control study.

In the present study, for both CRAO and noncentral RAO, the incidence was higher in men than in women; however, among those in their 20s, RAO incidences were higher in women. In Korea, RAO after cosmetic facial filler injection has been reported, and most of the cases were women in their 20s.^39,40^ Thus, it is likely that this iatrogenic RAO may explain the difference in the male-to-female ratio of RAO incidence in this younger age group.

RAO is considered a type of ischemic stroke.^9,41^ Globally, there were 12.2 million incident strokes and 6.55 million deaths from stroke in 2019,^41^ and stroke was the second-leading cause of death after ischemic heart disease.^41^ Stroke incidence varied greatly by country and region (eg, 92.2 to 232 per 100 000 person-years in Korea,^42^ 1.95 to 4.17 per 1000 person-years in China^43^).^41^ According to 1 report, the ischemic stroke in Korea was 92.2 per 100 000 person-years in men and 55.0 in women.^44^ Despite substantial variations, stroke incidence was higher than that of RAO and, like RAO, showed a tendency to increase with age.^34^ Moreover, studies have reported SMR similar to our study, and SMR showed a higher value for RAO than for stroke.^45,46^ In a Korean study, SMR of stroke was 2.26 (95% CI, 2.08–2.45) for 3 years.^45^ In a study of 17 149 patients with ischemic stroke in Sweden, the SMR was 5.88 (95% CI, 5.10-6.71) for men and 5.91 (CI, 4.68-7.29) for women, suggesting that the mortality of ischemic stroke was 6 times higher than that of the general population.^46^

In 2 previous Korean studies,^14,18^ there were no cases of GCA; however, the present study identified a total of 33 such cases. This may be due to the longer period in the present study (17 years) than previous studies. GCA was identified not only in CRAO (13 cases), but also in noncentral RAO (20 cases). The code for noncentral RAO (H34.2) also includes cilioretinal artery occlusion disorders. Cases with both noncentral RAO and GCA codes are probably cases of GCA with cilioretinal artery occlusion, not BRAO. Branch retinal arteries are in fact arterioles, not arteries, and GCA is a disease of medium and large arteries, not arterioles.^2^ Nevertheless, cilioretinal artery occlusion in GCA could be diagnosed erroneously as BRAO.^2,47^ In the present study, GCA was excluded to obtain only the incidence of nonarteritic RAO. Because GCA is a very rare disease, additional epidemiologic studies targeting GCA in a large population are needed.

### Limitations and Strengths

This study has some limitations. First, the incidence rate of RAO could be underestimated because of asymptomatic patients or patients who did not use health care services, which is a drawback of any study using a claims database. Second, the accuracy of diagnostic codes was not validated through medical records; thus, RAO incidence could be overestimated or underestimated. Non-RAO cases may have been classified as RAO cases; conversely, RAO may have been classified with a nonspecific diagnostic code or as RVO. However, since RAO is a very rare disease,^14^ it has a specific diagnostic code; accordingly, the possibility of misdiagnosis is thought to be low. Third, although most noncentral RAO cases may be BRAOs, as cilioretinal RAOs are thought to be very rare,^16^ it was impossible to accurately determine the proportion of BRAO among noncentral RAOs. Nevertheless, in the present study, the incidence and mortality rates of noncentral RAO and CRAO could be independently determined. Fourth, the incidence of stroke or cardiovascular diseases related to RAO could not be analyzed. In addition, we did not analyze comorbidities considering aging and incident comorbidities. However, comorbidity at the time of RAO diagnosis was investigated and compared between CRAO and noncentral RAO. Future large-population studies should investigate the risk of developing stroke or cardiovascular diseases in patients with newly diagnosed RAO. Also, we were unable to conduct an analysis that took competing risk of death into account.

Despite these limitations, our study has the advantage of being a large-scale epidemiological study in an Asian population, whereas there are many RAO-related studies targeting Western populations.^48,49^ Additionally, the incidence rate of RAO, including noncentral RAO, which is not well known, was reported, and the mortality rate of RAO compared with the general population was also presented.

## Conclusions

This cohort study investigated the incidence, comorbidity, mortality, and causes of death of clinically diagnosed RAO in Korea using population-based data. Although the comorbidities were similar, the incidence of noncentral RAO was higher than that of CRAO in Korea. Mortality among patients with RAO was significantly (about 7 times) higher than that in the general population. The most common causes of death were diseases of the circulatory system, such as acute myocardial infarction, suggesting the need for further research investigating the risk of developing cardiovascular or cerebrovascular disease in patients with newly diagnosed RAO.

## Notes

Supplement 1.
eMethods.
**eTable 1.** Frequencies and Incidence Rates of Retinal Artery Occlusion According to Sex **eTable 2.** Frequencies and Incidence Rates of Central Retinal Artery Occlusion Among Residents of Korea From 2005 to 2018 **eTable 3.** Frequencies and Incidence Rates of Non-Central Retinal Artery Occlusion Among Residents of Korea From 2005 to 2018 **eTable 4.** Mortality and SMRs of Retinal Artery Occlusion in Korea From 2005 to 2018 **eTable 5.** Mortality and SMRs of Central Retinal Artery Occlusion in Korea From 2005 to 2018 **eTable 6.** Mortality and SMRs of Non-Central Retinal Artery Occlusion in Korea From 2005 to 2018 **eTable 7.** Top 3 Causes of Death Among Patients With Retinal Artery Occlusion (2005-2018) and the General Population (2015) of Korea **eTable 8.** Causes of Death Among Patients With Retinal Artery Occlusion by Sex in Korea From 2005 to 2018 **eTable 9.** Top 3 Specific Causes of Death Among Patients With Retinal Artery Occlusion in Korea From 2005 to 2018 **eTable 10.** Causes of Death Among Patients With Retinal Artery Occlusion Aged <50 Years in Korea From 2005 to 2018

Supplement 2.
Data Sharing Statement
