# Supplementary material for: Incidence of Retinal Artery Occlusion and Related Mortality in Korea, 2005 to 2018
Source: JAMA Netw Open. 2023 Mar 10;6(3):e233068. doi: 10.1001/jamanetworkopen.2023.3068 (PMC12527418; doi:10.1001/jamanetworkopen.2023.3068)
Supplement: Supplement 1. — eMethods. eTable 1. Frequencies and Incidence Rates of Retinal Artery Occlusion According to Sex eTable 2. Frequencies and Incidence Rates of Central Retinal Artery Occlusion Among Residents of Korea From 2005 to 2018 eTable 3. Frequencies and Incidence Rates of Non-Central Retinal Artery Occlusion Among Residents of Korea From 2005 to 2018 eTable 4. Mortality and SMRs of Retinal Artery Occlusion in Korea From 2005 to 2018 eTable 5. Mortality and SMRs of Central Retinal Artery Occlusion in Korea From 2005 to 2018 eTable 6. Mortality and SMRs of Non-Central Retinal Artery Occlusion in Korea From 2005 to 2018 eTable 7. Top 3 Causes of Death Among Patients With Retinal Artery Occlusion (2005-2018) and the General Population (2015) of Korea eTable 8. Causes of Death Among Patients With Retinal Artery Occlusion by Sex in Korea From 2005 to 2018 eTable 9. Top 3 Specific Causes of Death Among Patients With Retinal Artery Occlusion in Korea From 2005 to 2018 eTable 10. Causes of Death Among Patients With Retinal Artery Occlusion Aged <50 Years in Korea From 2005 to 2018 [file jamanetwopen-e233068-s001.pdf]

Supplemental Online Content

Hwang DDJ, Lee KE, Kim Y, et al. Incidence of retinal artery occlusion and related mortality in Korea, 2005 to 2018. *JAMA Netw Open.* 2023;6(3):e233068. doi:10.1001/jamanetworkopen.2023.3068

eMethods.

eTable 1. Frequencies and Incidence Rates of Retinal Artery Occlusion According to Sex

eTable 2. Frequencies and Incidence Rates of Central Retinal Artery Occlusion Among Residents of Korea From 2005 to 2018

eTable 3. Frequencies and Incidence Rates of Non-Central Retinal Artery Occlusion Among Residents of Korea From 2005 to 2018

eTable 4. Mortality and SMRs of Retinal Artery Occlusion in Korea From 2005 to 2018

eTable 5. Mortality and SMRs of Central Retinal Artery Occlusion in Korea From 2005 to 2018

eTable 6. Mortality and SMRs of Non-Central Retinal Artery Occlusion in Korea From 2005 to 2018

eTable 7. Top 3 Causes of Death Among Patients With Retinal Artery Occlusion (2005-2018) and the General Population (2015) of Korea

eTable 8. Causes of Death Among Patients With Retinal Artery Occlusion by Sex in Korea From 2005 to 2018

eTable 9. Top 3 Specific Causes of Death Among Patients With Retinal Artery Occlusion in Korea From 2005 to 2018

eTable 10. Causes of Death Among Patients With Retinal Artery Occlusion Aged <50 Years in Korea From 2005 to 2018

This supplemental material has been provided by the authors to give readers additional information about their work.

© 2023 Hwang DDJ et al. *JAMA Network Open.*

eMethods

Study Population

The NHIS database includes records for the entire Korean population, as a single-payer health insurance program covers every resident of Korea through either the National Health Insurance system (97% of residents), which is a compulsory health insurance, or the Medical Aid system (3% of residents).[17] Thus, the NHIS database contains all medical records associated with medical insurance claims (e.g., diagnoses, procedures, prescription drugs used, demographic information, and direct medical costs) in Korea. All patients in the NHIS database are identified by their Korean Resident Registration Number, a unique identification number assigned to each Korean resident at birth, which ensures no duplications or omissions when accessing the data.

Definition of Retinal Artery Occlusion

CRAO/non-central RAO cases were identified according to the first RAO diagnostic code (H34.1[14,18] for CRAO and H34.2[5] for non-central RAO, i.e. other RAO) in the Korean Classification of Disease, seventh edition, a version of the International Classification of Diseases (ICD), 10th edition, adapted for the Korean healthcare system. The probable diagnosis of non-central RAO mainly includes branch RAO (BRAO) and cilioretinal artery occlusion disorders. The date of the earliest claim associated with the ICD-10 diagnostic code for RAO in the NHIS database was defined as the index date and was considered as the time of disease incidence.

Mortality and Cause of Death

To determine mortality and causes of death, we linked the NHIS data to Statistics Korea data. Statistics Korea is a government operated database established in 1981 that includes death certificates, confirmed by physicians, of all deceased persons in Korea. The cause of death was investigated for all RAO mortality cases using Statistics Korea data from 2005 to 2018. The cause of death was coded and classified as per the ICD-10 code.

Comorbidities

Presence of hypertension, dyslipidemia, diabetes mellitus, ischemic heart disease, ischemic stroke, heart failure, chronic kidney disease, arterial fibrillation and flutter, hemorrhagic stroke, cardiac valvular disorders were defined based on ICD-10 diagnostic codes provided in previous reports.[21]

© 2023 Hwang DDJ et al. *JAMA Network Open.*

eTable 1. Frequencies and incidence rates of retinal artery occlusion according to sex

Age (yrs)	General Population[a]			Incidence[b]				Male-to-Female Ratio (P-value[c])
	Total	Men	Women	Men		Women		
				N	Incidence (95% CI)	N	Incidence (95% CI)	
<5	2235397	1147126	1088271	10	0·06 (0·03-0·12)	11	0·07 (0·04-0·13)	0·86 (0·7346)
5-9	2252950	1162087	1090863	13	0·08 (0·04-0·14)	9	0·06 (0·03-0·11)	1·36 (0·4809)
10-14	2418360	1257902	1160458	54	0·31 (0·23-0·40)	30	0·19 (0·13-0·26)	1·66 (0·0244)
15-19	3170545	1657722	1512823	143	0·62 (0·52-0·73)	75	0·35 (0·28-0·44)	1·74 (0·0001)
20-24	3385936	1808857	1577079	172	0·68 (0·58-0·79)	194	0·88 (0·76-1·01)	0·77 (0·0137)
25-29	3027896	1581887	1446009	277	1·25 (1·11-1·41)	269	1·33 (1·18-1·50)	0·94 (0·4796)
30-34	3611034	1854905	1756129	430	1·66 (1·50-1·82)	280	1·14 (1·01-1·28)	1·45 (0·0000)
35-39	3783589	1927388	1856201	638	2·37 (2·19-2·56)	484	1·86 (1·70-2·04)	1·27 (<·0001)
40-44	4215921	2142101	2073820	1087	3·63 (3·41-3·85)	756	2·60 (2·42-2·80)	1·39 (<·0001)
45-49	4266941	2151070	2115871	1650	5·48 (5·22-5·75)	1264	4·27 (4·04-4·51)	1·28 (<·0001)
50-54	4145976	2094318	2051658	2306	7·87 (7·55-8·20)	1853	6·45 (6·16-6·75)	1·22 (<·0001)
55-59	3863095	1922796	1940299	3066	11·40 (11·00-11·81)	2417	8·90 (8·55-9·26)	1·28 (<·0001)
60-64	2758941	1348273	1410668	3740	19·84 (19·21-20·48)	2628	13·32 (12·81-13·84)	1·49 (<·0001)
65-69	2117875	1015463	1102412	4875	34·35 (33·40-35·33)	3158	20·49 (19·78-21·21)	1·68 (<·0001)
70-74	1760932	789607	971325	4757	43·12 (41·90-44·36)	3247	23·91 (23·10-24·75)	1·81 (<·0001)
75-79	1356014	550684	805330	3025	39·31 (37·92-40·74)	2997	26·62 (25·67-27·59)	1·48 (<·0001)
80-84	810891	275462	535429	1739	45·19 (43·09-47·36)	1864	24·90 (23·78-26·05)	1·82 (<·0001)
85-89	371527	98367	273160	733	53·34 (49·55-57·35)	777	20·34 (18·93-21·82)	2·63 (<·0001)
90-94	124111	28565	95546	126	31·55 (26·28-37·56)	137	10·25 (8·60-12·11)	3·09 (<·0001)
≥95	27732	5259	22473	16	21·75 (12·43-35·32)	19	6·04 (3·64-9·43)	3·61 (<·0001)
Total	49705663	24819839	24885824	28857	8·31 (8·21-8·41)	22469	6·45 (6·37-6·54)	1·29 (<·0001)

[a] Information on the Korean population was based on 2015 census data from the Korean Statistical Information Service.
[b] Incidence rate per 100,000 person-years.
[c] P-value, as determined by the chi-square test.

© 2023 Hwang DDJ et al. *JAMA Network Open.*

eTable 2. Frequencies and incidence rates of central retinal artery occlusion among residents of Korea from 2005 to 2018[a]

Age (yrs)	Incidence[b]						Male-to-Female Ratio (P-value[c])
	Total		Male		Female		
	N	Incidence(95% CI)	N	Incidence(95% CI)	N	Incidence(95% CI)	
<5	16	0.05 (0.03-0.08)	9	0.06 (0.03-0.11)	7	0.05 (0.02-0.10)	1.22 (0.6930)
5-9	7	0.02 (0.01-0.05)	5	0.03 (0.01-0.07)	2	0.01 (0.00-0.05)	2.35 (0.2934)
10-14	24	0.07 (0.05-0.11)	18	0.10 (0.06-0.16)	6	0.04 (0.01-0.08)	2.77 (0.0242)
15-19	75	0.17 (0.13-0.21)	50	0.22 (0.16-0.28)	25	0.12 (0.08-0.17)	1.83 (0.0126)
20-24	116	0.25 (0.20-0.29)	49	0.19 (0.14-0.26)	67	0.30 (0.24-0.39)	0.64 (0.0158)
25-29	206	0.49 (0.42-0.56)	111	0.50 (0.41-0.60)	95	0.47 (0.38-0.57)	1.07 (0.6375)
30-34	277	0.55 (0.49-0.62)	160	0.62 (0.52-0.72)	117	0.48 (0.39-0.57)	1.29 (0.0332)
35-39	374	0.71 (0.64-0.78)	214	0.79 (0.69-0.91)	160	0.62 (0.52-0.72)	1.29 (0.0151)
40-44	618	1.05 (0.97-1.13)	387	1.29 (1.17-1.43)	231	0.80 (0.70-0.91)	1.62 (<.0001)
45-49	881	1.48 (1.38-1.58)	544	1.81 (1.66-1.97)	337	1.14 (1.02-1.27)	1.59 (<.0001)
50-54	1171	2.02 (1.90-2.14)	726	2.48 (2.30-2.66)	445	1.55 (1.41-1.70)	1.60 (<.0001)
55-59	1457	2.69 (2.56-2.84)	896	3.33 (3.12-3.56)	561	2.07 (1.90-2.24)	1.61 (<.0001)
60-64	1724	4.47 (4.26-4.68)	1119	5.93 (5.59-6.29)	605	3.06 (2.83-3.32)	1.94 (<.0001)
65-69	2269	7.66 (7.35-7.98)	1416	9.97 (9.45-10.50)	853	5.53 (5.16-5.91)	1.80 (<.0001)
70-74	2427	9.85 (9.46-10.25)	1446	13.09 (12.43-13.78)	981	7.22 (6.77-7.68)	1.81 (<.0001)
75-79	2073	10.93 (10.46-11.41)	1137	14.76 (13.92-15.64)	936	8.31 (7.78-8.86)	1.78 (<.0001)
80-84	1292	11.39 (10.78-12.03)	635	16.48 (15.22-17.81)	657	8.77 (8.11-9.47)	1.88 (<.0001)
85-89	554	10.66 (9.79-11.58)	239	17.37 (15.24-19.72)	315	8.24 (7.36-9.20)	2.11 (<.0001)
90-94	112	6.45 (5.31-7.76)	53	13.26 (9.93-17.35)	59	4.41 (3.36-5.69)	3.01 (<.0001)
≥95	11	2.83 (1.42-5.07)	6	8.15 (2.99-17.74)	5	1.59 (0.52-3.71)	5.13 (0.0026)
Total	15684	2.25 (2.22-2.29)	9220	2.65 (2.60-2.71)	6464	1.86 (1.81-1.90)	1.43 (<.0001)

[a] Information on the Korean population was based on 2015 census data from the Korean Statistical Information Service.
[b] Incidence rate was measured as cases per 100,000 person-years.
[c] P-value, as determined by the chi-square test.
CI = confidence interval

© 2023 Hwang DDJ et al. *JAMA Network Open.*

eTable 3. Frequencies and incidence rates of non-central retinal artery occlusion among residents of Korea from 2005 to 2018[a]

Age (yrs)	Incidence[b]						Male-to-Female Ratio (P-value[c])
	Total		Male		Female		
	N	Incidence(95% CI)	N	Incidence(95% CI)	N	Incidence(95% CI)	
<5	5	0.02 (0.01-0.04)	1	0.01 (0.00-0.04)	4	0.03 (0.01-0.07)	0.24 (0.1612)
5-9	15	0.05 (0.03-0.08)	8	0.05 (0.02-0.10)	7	0.05 (0.02-0.09)	1.07 (0.8920)
10-14	60	0.18 (0.14-0.23)	36	0.20 (0.14-0.28)	24	0.15 (0.10-0.22)	1.38 (0.2157)
15-19	143	0.32 (0.27-0.38)	93	0.40 (0.32-0.49)	50	0.24 (0.18-0.31)	1.70 (0.0023)
20-24	250	0.53 (0.46-0.60)	123	0.49 (0.40-0.58)	127	0.58 (0.48-0.68)	0.84 (0.1807)
25-29	340	0.80 (0.72-0.89)	166	0.75 (0.64-0.87)	174	0.86 (0.74-1.00)	0.87 (0.2067)
30-34	433	0.86 (0.78-0.94)	270	1.04 (0.92-1.17)	163	0.66 (0.57-0.77)	1.57 (<.0001)
35-39	748	1.41 (1.31-1.52)	424	1.57 (1.43-1.73)	324	1.25 (1.12-1.39)	1.26 (0.0017)
40-44	1225	2.08 (1.96-2.20)	700	2.33 (2.17-2.51)	525	1.81 (1.66-1.97)	1.29 (<.0001)
45-49	2033	3.40 (3.26-3.56)	1106	3.67 (3.46-3.90)	927	3.13 (2.93-3.34)	1.17 (0.0003)
50-54	2988	5.15 (4.97-5.34)	1580	5.39 (5.13-5.66)	1408	4.90 (4.65-5.17)	1.10 (0.0097)
55-59	4026	7.45 (7.22-7.68)	2170	8.06 (7.73-8.41)	1856	6.84 (6.53-7.15)	1.18 (<.0001)
60-64	4644	12.03 (11.69-12.38)	2621	13.90 (13.37-14.44)	2023	10.25 (9.81-10.71)	1.36 (<.0001)
65-69	5764	19.46 (18.96-19.97)	3459	24.36 (23.55-25.18)	2305	14.95 (14.34-15.57)	1.63 (<.0001)
70-74	5577	22.65 (22.06-23.25)	3311	29.99 (28.98-31.03)	2266	16.68 (16.00-17.38)	1.80 (<.0001)
75-79	3949	20.82 (20.18-21.48)	1888	24.52 (23.42-25.65)	2061	18.30 (17.52-19.10)	1.34 (<.0001)
80-84	2311	20.38 (19.55-21.22)	1104	28.66 (27.00-30.40)	1207	16.11 (15.22-17.05)	1.78 (<.0001)
85-89	956	18.39 (17.25-19.60)	494	35.92 (32.82-39.23)	462	12.09 (11.01-13.24)	2.98 (<.0001)
90-94	151	8.69 (7.36-10.20)	73	18.26 (14.32-22.96)	78	5.83 (4.61-7.28)	3.14 (<.0001)
≥95	24	6.18 (3.96-9.20)	10	13.59 (6.52-24.99)	14	4.45 (2.43-7.47)	3.06 (0.0045)
Total	35642	5.12 (5.07-5.18)	19637	5.65 (5.57-5.73)	16005	4.60 (4.52-4.67)	1.23 (<.0001)

[a] Information on the Korean population was based on 2015 census data from the Korean Statistical Information Service.
[b] Incidence rate was measured as cases per 100,000 person-years.
[c] P-value, as determined by the chi-square test.
CI= confidence interval

© 2023 Hwang DDJ et al. *JAMA Network Open.*

eTable 4. Mortality and SMRs of retinal artery occlusion in Korea from 2005 to 2018

Age Group (yrs)	All Participants			Male			Female		
	No· of Observed Deaths	No· of Expected Deaths	SMR (95% CI)	No· of Observed Deaths	No· of Expected Deaths	SMR (95% CI)	No· of Observed Deaths	No· of Expected Deaths	SMR (95% CI)
Total	7107	277·3	7·33 (7·15-7·50)	4549	169·5	6·99 (6·79-7·20)	2558	110·7	8·00 (7·69-8·31)
<5	1	0·01	72·82 (3·64-359·10)	1	0·01	140·83 (7·05-694·50)	0	0·01	-
5-9	0	0·00	-	0	0·00	-	0	0·00	-
10-14	0	0·01	-	0	0·01	-	0	0·00	-
15-19	3	0·05	64·17 (16·32-174·60)	2	0·04	52·09 (8·73-172·10)	1	0·01	85·82 (4·29-423·20)
20-24	4	0·12	33·41 (10·62-80·60)	3	0·07	41·78 (10·63-113·70)	1	0·04	23·08 (1·16-113·80)
25-29	14	0·25	56·61 (26·96-86·26)	10	0·16	63·30 (24·07-102·54)	4	0·09	46·34 (·93-91·76)
30-34	19	0·43	44·06 (24·25-63·87)	14	0·31	45·81 (21·81-69·81)	5	0·14	36·09 (4·46-67·72)
35-39	31	0·89	34·96 (22·66-47·27)	21	0·63	33·59 (19·22-47·95)	10	0·28	35·10 (13·34-56·86)
40-44	67	2·35	28·49 (21·67-35·31)	46	1·83	25·16 (17·89-32·43)	21	0·64	32·61 (18·66-46·56)
45-49	129	5·73	22·53 (18·64-26·41)	100	4·57	21·88 (17·59-26·17)	29	1·45	20·06 (12·76-27·36)
50-54	225	12·30	18·29 (15·90-20·68)	170	10·05	16·92 (14·38-19·46)	55	2·83	19·40 (14·28-24·53)
55-59	373	22·37	16·67 (14·98-18·37)	274	18·70	14·65 (12·92-16·39)	99	5·03	19·69 (15·81-23·57)
60-64	569	37·68	15·10 (13·86-16·34)	434	33·18	13·08 (11·85-14·31)	135	8·12	16·62 (13·81-19·42)
65-69	959	74·56	12·86 (12·05-13·68)	703	66·87	10·51 (9·74-11·29)	256	16·41	15·60 (13·69-17·52)
70-74	1450	137·41	10·55 (10·01-11·10)	962	117·81	8·17 (7·65-8·68)	488	35·69	13·67 (12·46-14·89)
75-79	1440	188·55	7·64 (7·24-8·03)	851	134·83	6·31 (5·89-6·74)	589	66·68	8·83 (8·12-9·55)
80-84	1128	199·26	5·66 (5·33-5·99)	618	131·47	4·70 (4·33-5·07)	510	83·62	6·10 (5·57-6·63)

© 2023 Hwang DDJ et al. *JAMA Network Open.*

85-89	565	153·00	3·69 (3·39-4·00)	284	97·88	2·90 (2·56-3·24)	281	69·70	4·03 (3·56-4·50)
≥90	130	58·17	2·23 (1·85-2·62)	56	31·99	1·75 (1·29-2·21)	74	29·10	2·54 (1·96-3·12)

SMR = standardized mortality ratio; CI = confidence interval

eTable 5. Mortality and SMRs of central retinal artery occlusion in Korea from 2005 to 2018

Age Group (yrs)	CRAO			Male			Female		
	No. of Observed Deaths	No. of Expected Deaths	SMR (95% CI)	No. of Observed Deaths	No. of Expected Deaths	SMR (95% CI)	No. of Observed Deaths	No. of Expected Deaths	SMR (95% CI)
Total	3289	84.7	9.95 (9.61-10.29)	2134	54.2	9.69 (9.28-10.10)	1155	31.8	10.48 (9.87-11.08)
<5	1	0.01	95.58 (4.78-471.40)	1	0.01	156.48 (7.83-771.70)	0	0.00	..
5-9	0	0.00	..	0	0.00	..	0	0.00	..
10-14	0	0.00	..	0	0.00	..	0	0.00	..
15-19	2	0.02	124.34 (20.85-410.80)	1	0.01	74.49 (3.73-367.40)	1	0.00	257.45 (12.88-1270.00)
20-24	3	0.04	79.07 (20.11-215.20)	2	0.02	97.78 (16.39-323.10)	1	0.01	66.83 (3.34-329.60)
25-29	10	0.09	107.17 (40.75-173.60)	7	0.06	110.58 (28.66-192.49)	3	0.03	98.41 (25.03-267.80)
30-34	9	0.17	53.50 (18.55-88.45)	7	0.11	61.56 (15.96-107.16)	2	0.06	34.54 (5.79-114.10)
35-39	10	0.30	33.84 (12.86-54.81)	6	0.21	28.61 (5.72-51.50)	4	0.09	42.47 (0.85-84.09)
40-44	30	0.79	38.04 (24.43-51.65)	17	0.65	26.12 (13.70-38.54)	13	0.20	66.07 (30.16-101.99)
45-49	55	1.73	31.77 (23.37-40.16)	44	1.51	29.20 (20.57-37.83)	11	0.39	28.54 (11.67-45.41)
50-54	104	3.46	30.03 (24.26-35.80)	77	3.16	24.34 (18.91-29.78)	27	0.68	39.66 (24.70-54.63)
55-59	157	5.94	26.41 (22.28-30.54)	119	5.46	21.78 (17.86-25.69)	38	1.17	32.57 (22.21-42.92)

60-64	240	10.20	23.53 (20.55-26.50)	186	9.93	18.74 (16.04-21.43)	54	1.87	28.87 (21.17-36.57)
65-69	451	21.06	21.42 (19.44-23.39)	335	19.42	17.25 (15.40-19.09)	116	4.43	26.18 (21.41-30.94)
70-74	666	41.67	15.98 (14.77-17.20)	466	35.81	13.01 (11.83-14.19)	200	10.78	18.55 (15.98-21.12)
75-79	681	64.91	10.49 (9.70-11.28)	426	50.68	8.41 (7.61-9.20)	255	20.82	12.25 (10.74-13.75)
80-84	525	71.45	7.35 (6.72-7.98)	279	48.01	5.81 (5.13-6.49)	246	29.47	8.35 (7.30-9.39)
85-89	279	56.13	4.97 (4.39-5.55)	131	31.91	4.10 (3.40-4.81)	148	28.26	5.24 (4.39-6.08)
≥90	66	24.01	2.75 (2.09-3.41)	30	13.29	2.26 (1.45-3.06)	36	11.94	3.02 (2.03-4.00)

CI = confidence interval, CRAO = central retinal artery occlusion, SMR = standardized mortality ratio

© 2023 Hwang DDJ et al. *JAMA Network Open.*

eTable 6. Mortality and SMRs of non-central retinal artery occlusion in Korea from 2005 to 2018

Age Group (yrs)	Non-central RAO			Male			Female		
	No. of Observed Deaths	No. of Expected Deaths	SMR (95% CI)	No. of Observed Deaths	No. of Expected Deaths	SMR (95% CI)	No. of Observed Deaths	No. of Expected Deaths	SMR (95% CI)
Total	3818	192.6	5.97 (5.78-6.16)	2415	115.4	5.61 (5.39-5.84)	1403	78.9	6.69 (6.34-7.04)
<5	0	0.00	..	0	0.00	..	0	0.00	..
5-9	0	0.00	..	0	0.00	..	0	0.00	..
10-14	0	0.01	..	0	0.00	..	0	0.00	..
15-19	1	0.03	32.61 (1.63-160.80)	1	0.02	40.05 (2.00-197.50)	0	0.01	..
20-24	1	0.08	12.23 (0.61-60.31)	1	0.05	19.48 (0.97-96.06)	0	0.03	..
25-29	4	0.15	25.97 (0.52-51.43)	3	0.09	31.69 (8.06-86.24)	1	0.06	17.91 (.90-88.33)
30-34	10	0.26	38.03 (14.46-61.59)	7	0.19	36.48 (9.46-63.50)	3	0.08	37.19 (9.46-101.20)
35-39	21	0.59	35.53 (20.33-50.72)	15	0.42	36.10 (17.83-54.37)	6	0.19	31.46 (6.29-56.63)
40-44	37	1.56	23.67 (16.04-31.29)	29	1.18	24.63 (15.67-33.60)	8	0.45	17.89 (5.49-30.29)
45-49	74	4.00	18.52 (14.30-22.74)	56	3.06	18.28 (13.49-23.07)	18	1.06	16.98 (9.13-24.82)
50-54	121	8.84	13.69 (11.25-16.13)	93	6.88	13.51 (10.76-16.26)	28	2.15	13.00 (8.19-17.82)
55-59	216	16.43	13.15 (11.40-14.90)	155	13.23	11.71 (9.87-13.56)	61	3.86	15.80 (11.84-19.77)
60-64	329	27.48	11.97 (10.68-13.27)	248	23.25	10.67 (9.34-11.99)	81	6.25	12.95 (10.13-15.77)
65-69	508	53.50	9.50 (8.67-10.32)	368	47.45	7.76 (6.96-8.55)	140	11.97	11.69 (9.75-13.63)
70-74	784	95.74	8.19 (7.62-8.76)	496	82.00	6.05 (5.52-6.58)	288	24.91	11.56 (10.23-12.90)
75-79	759	123.65	6.14 (5.70-6.58)	425	84.15	5.05 (4.57-5.53)	334	45.85	7.28 (6.50-8.07)
80-84	603	127.81	4.72 (4.34-5.09)	339	83.46	4.06 (3.63-4.49)	264	54.15	4.88 (4.29-5.46)
85-89	286	96.86	2.95 (2.61-3.29)	153	65.96	2.32 (1.95-2.69)	133	41.45	3.21 (2.66-3.75)
≥90	64	34.16	1.87 (1.41-2.33)	26	18.70	1.39 (0.86-1.93)	38	17.16	2.21 (1.51-2.92)

CI = confidence interval, RAO = retinal artery occlusion, SMR = standardized mortality ratio

© 2023 Hwang DDJ et al. *JAMA Network Open.*

eTable 7. Top 3 causes of death among patients with retinal artery occlusion (2005-2018) and the general population (2015) of Korea

	**RAO**<br>N = 7053	
	**Causes of Death (ICD-10 Code)**	**Number of Cases (%)**
1st	Diseases of the circulatory system (I00-I99)	2,029 (28·8)
2nd	Neoplasms (C00-D48)	1,768 (25·1)
3rd	Diseases of the respiratory system (J00-J98,U04)	721 (10·2)
	**RAO under aged 50**<br>N = 258	
	**Causes of Death (ICD-10 Code)**	**Number of Cases (%)**
1st	Neoplasms (C00-D48)	60 (22·4)
2nd	Diseases of the circulatory system (I00-I99)	59 (22·0)
3rd	External causes of morbidity (V01-Y89)[a]	41 (15·3)
	**General population**<br>N = 275,895	
	**Causes of Death (ICD-10 Code)**	**Number of Cases (%)**
1st	Neoplasms (C00-D48)	78,281 (28·4)
2nd	Diseases of the circulatory system (I00-I99)	59,543 (21·6)
3rd	External causes of morbidity (V01-Y89)[b]	28,784 (10·4)
	**General population under aged 50**<br>N = 27,589	
	**Causes of Death (ICD-10 Code)**	**Number of Cases (%)**
1st	External causes of morbidity (V01-Y89)[c]	9,306 (37·5)
2nd	Neoplasms (C00-D48)	5,969 (24·1)
3rd	Diseases of the circulatory system (I00-I99)	2,869 (11·6)

[a] There were no cases with Suicide (X60–X84) codes.

[b] There were 13513 cases with Suicide (X60–X84) codes.

[c] There were 5882 cases with Suicide (X60–X84) codes.

RAO = retinal artery occlusion; ICD = International Classification of Diseases

© 2023 Hwang DDJ et al. *JAMA Network Open.*

eTable 8. Causes of death among patients with retinal artery occlusion by sex in Korea from 2005 to 2018

Causes of Death (ICD-10 Code)	Number of Cases (%)								
	RAO			CRAO			Non-central RAO		
	Total	Men	Women	Total	Men	Women	Total	Men	Women
	7053	4522	2531	3269	2125	1144	3784	2397	1387
Diseases of the circulatory system (I00-I99)	**2029 (28.8%)**	**1218 (26.9%)**	**811 (32.0%)**	**962 (29.4%)**	**574 (27.0%)**	**388 (33.9%)**	**1067 (28.2%)**	**644 (26.9%)**	**423 (30.5%)**
Neoplasms (C00-D48)	**1768 (25.1%)**	**1275 (28.2%)**	**493 (19.5%)**	**795 (24.3%)**	**586 (27.6%)**	**209 (18.3%)**	**973 (25.7%)**	**689 (28.7%)**	**284 (20.5%)**
Diseases of the respiratory system (J00-J98,U04)	**721 (10.2%)**	**509 (11.3%)**	**212 (8.4%)**	**333 (10.2%)**	**245 (11.5%)**	**88 (7.7%)**	**388 (10.3%)**	**264 (11.0%)**	**124 (8.9%)**
Endocrine, nutritional and metabolic diseases (E00-E88)	524 (7.4%)	346 (7.7%)	178 (7.0%)	265 (8.1%)	177 (8.3%)	**88 (7.7%)**	259 (6.8%)	169 (7.1%)	90 (6.5%)
Symptoms, signs and abnormal clinical and laboratory findings, not elsewhere classified (R00-R99)	506 (7.2%)	276 (6.1%)	230 (9.1%)	232 (7.1%)	133 (6.3%)	99 (8.7%)	274 (7.2%)	143 (6.0%)	131 (9.4%)
External causes of morbidity (V01-Y89)	465 (6.6%)	319 (7.1%)	146 (5.8%)	208 (6.4%)	148 (7.0%)	60 (5.2%)	257 (6.8%)	171 (7.1%)	86 (6.2%)
Diseases of the genitourinary system (N00-N98)	274 (3.9%)	160 (3.5%)	114 (4.5%)	118 (3.6%)	67 (3.2%)	51 (4.5%)	156 (4.1%)	93 (3.9%)	63 (4.5%)
Certain infectious and parasitic diseases (A00-B99)	215 (3.0%)	126 (2.8%)	89 (3.5%)	96 (2.9%)	52 (2.4%)	44 (3.8%)	119 (3.1%)	74 (3.1%)	45 (3.2%)
Diseases of the digestive system (K00-K92)	206 (2.9%)	131 (2.9%)	75 (3.0%)	95 (2.9%)	57 (2.7%)	38 (3.3%)	111 (2.9%)	74 (3.1%)	37 (2.7%)
Diseases of the nervous system (G00-G98)	163 (2.3%)	78 (1.7%)	85 (3.4%)	67 (2.0%)	30 (1.4%)	37 (3.2%)	96 (2.5%)	48 (2.0%)	48 (3.5%)
Mental, Behavioral and Neurodevelopmental disorders (F01-F99)	88 (1.2%)	38 (0.8%)	50 (2.0%)	47 (1.4%)	26 (1.2%)	21 (1.8%)	41 (1.1%)	12 (0.5%)	29 (2.1%)
Diseases of the musculoskeletal system and connective tissue (M00-M99)	56 (0.8%)	23 (0.5%)	33 (1.3%)	31 (0.9%)	14 (0.7%)	17 (1.5%)	25 (0.7%)	9 (0.4%)	16 (1.2%)
Diseases of the blood and blood-forming organs and certain disorders involving the immune mechanism (D50-D89)	17 (0.2%)	8 (0.2%)	9 (0.4%)	7 (0.2%)	4 (0.2%)	3 (0.3%)	10 (0.3%)	4 (0.2%)	6 (0.4%)
Diseases of the skin and subcutaneous tissue (L00-L98)	13 (0.2%)	9 (0.2%)	4 (0.2%)	6 (0.2%)	6 (0.3%)	0 (0.0%)	7 (0.2%)	3 (0.1%)	4 (0.3%)
Congenital malformations, deformations and chromosomal abnormalities (Q00-Q99)	5 (0.1%)	4 (0.1%)	1 (0.0%)	5 (0.2%)	4 (0.2%)	1 (0.1%)	0 (0.0%)	0 (0.0%)	0 (0.0%)
Diseases of the eye and adnexa (H00-H57)	2 (0.0%)	2 (0.0%)	0 (0.0%)	2 (0.1%)	2 (0.1%)	0 (0.0%)	0 (0.0%)	0 (0.0%)	0 (0.0%)
Pregnancy, childbirth and the puerperium (O00-O99)	1 (0.0%)	0 (0.0%)	1 (0.0%)	0 (0.0%)	0 (0.0%)	0 (0.0%)	1 (0.0%)	0 (0.0%)	1 (0.1%)
Diseases of the ear and mastoid process (H60-H93)	0 (0.0%)	0 (0.0%)	0 (0.0%)	0 (0.0%)	0 (0.0%)	0 (0.0%)	0 (0.0%)	0 (0.0%)	0 (0.0%)
Certain conditions originating in the perinatal period (P00-P96)	0 (0.0%)	0 (0.0%)	0 (0.0%)	0 (0.0%)	0 (0.0%)	0 (0.0%)	0 (0.0%)	0 (0.0%)	0 (0.0%)

RAO = retinal artery occlusion; CRAO = central retinal artery occlusion; ICD = International Classification of Diseases

© 2023 Hwang DDJ et al. *JAMA Network Open.*

eTable 9. Top 3 specific causes of death among patients with RAO in Korea from 2005 to 2018

Causes of Death (ICD-10 Code)	Number of Cases (%)								
	RAO			CRAO			Non-central RAO		
	Total	Male	Female	Total	Male	Female	Total	Male	Female
	7053	4522	2531	3269	2125	1144	3784	2397	1387
**Diseases of the circulatory system (I00-I99)**	2029 (100.0%)	1218 (100.0%)	811 (100.0%)	962 (100.0%)	574 (100.0%)	388 (100.0%)	1067 (100.0%)	644 (100.0%)	423 (100.0%)
I21: Acute myocardial infarction	378 (18.6%)	242 (19.9%)	136 (16.8%)	184 (19.1%)	117 (20.4%)	67 (17.3%)	194 (18.2%)	125 (19.4%)	69 (16.3%)
I63: Cerebral infarction	322 (15.9%)	213 (17.5%)	109 (13.4%)	163 (16.9%)	116 (20.2%)	47 (12.1%)	159 (14.9%)	97 (15.1%)	62 (14.7%)
I69: Sequelae of cerebrovascular disease	247 (12.2%)	158 (13.0%)	89 (11.0%)	125 (13.0%)	76 (13.2%)	49 (12.6%)	122 (11.4%)	82 (12.7%)	40 (9.5%)
I61: Nontraumatic intracerebral hemorrhage	150 (7.4%)	88 (7.2%)	62 (7.6%)	70 (7.3%)	42 (7.3%)	28 (7.2%)	80 (7.5%)	46 (7.1%)	34 (8.0%)
I50: Heart failure	149 (7.3%)	79 (6.5%)	70 (8.6%)	65 (6.8%)	35 (6.1%)	30 (7.7%)	84 (7.9%)	44 (6.8%)	40 (9.5%)
Others	783 (38.6%)	438 (36.0%)	345 (42.5%)	355 (36.9%)	188 (32.8%)	167 (43.0%)	428 (40.1%)	250 (38.8%)	178 (42.1%)
**Neoplasms (C00-D48)**	1768 (100.0%)	1275 (100.0%)	493 (100.0%)	795 (100.0%)	586 (100.0%)	209 (100.0%)	973 (100.0%)	689 (100.0%)	284 (100.0%)
C34: Malignant neoplasm of bronchus and lung	470 (26.6%)	376 (29.5%)	94 (19.1%)	224 (28.2%)	184 (31.4%)	40 (19.1%)	246 (25.3%)	192 (27.9%)	54 (19.0%)
C22: Malignant neoplasm of liver and intrahepatic bile ducts	217 (12.3%)	166 (13.0%)	51 (10.3%)	89 (11.2%)	65 (11.1%)	24 (11.5%)	128 (13.2%)	101 (14.7%)	27 (9.5%)
C16: Malignant neoplasm of stomach	168 (9.5%)	130 (10.2%)	38 (7.7%)	73 (9.2%)	60 (10.2%)	13 (6.2%)	95 (9.8%)	70 (10.2%)	25 (8.8%)
C18: Malignant neoplasm of colon	99 (5.6%)	65 (5.1%)	34 (6.9%)	43 (5.4%)	33 (5.6%)	10 (4.8%)	56 (5.8%)	32 (4.6%)	24 (8.5%)
C25: Malignant neoplasm of pancreas	97 (5.5%)	64 (5.0%)	33 (6.7%)	38 (4.8%)	28 (4.8%)	10 (4.8%)	59 (6.1%)	36 (5.2%)	23 (8.1%)
Others	717 (40.6%)	474 (37.2%)	243 (49.3%)	328 (41.3%)	216 (36.9%)	112 (53.6%)	389 (40.0%)	258 (37.4%)	131 (46.1%)
**Diseases of the respiratory system (J00-J98,U04)**	721 (100.0%)	509 (100.0%)	212 (100.0%)	333 (100.0%)	245 (100.0%)	88 (100.0%)	388 (100.0%)	264 (100.0%)	124 (100.0%)
J18: Pneumonia, unspecified organism	335 (46.5%)	224 (44.0%)	111 (52.4%)	161 (48.3%)	117 (47.8%)	44 (50.0%)	174 (44.8%)	107 (40.5%)	67 (54.0%)
J44: Other chronic obstructive pulmonary disease	141 (19.6%)	114 (22.4%)	27 (12.7%)	60 (18.0%)	52 (21.2%)	8 (9.1%)	81 (20.9%)	62 (23.5%)	19 (15.3%)
J69: Pneumonitis due to solids and liquids	51 (7.1%)	38 (7.5%)	13 (6.1%)	24 (7.2%)	18 (7.3%)	6 (6.8%)	27 (7.0%)	20 (7.6%)	7 (5.6%)
J84: Other interstitial pulmonary diseases	40 (5.6%)	29 (5.7%)	11 (5.2%)	15 (4.5%)	12 (4.9%)	3 (3.4%)	25 (6.4%)	17 (6.4%)	8 (6.5%)
J45: Asthma	38 (5.3%)	20 (3.9%)	18 (8.5%)	21 (6.3%)	9 (3.7%)	12 (13.6%)	17 (4.4%)	11 (4.2%)	6 (4.8%)
Others	116 (16.1%)	84 (32.0%)	32 (15.1%)	52 (15.6%)	37 (15.1%)	15 (17.0%)	64 (16.5%)	47 (17.8%)	17 (13.7%)

RAO = retinal artery occlusion; CRAO = central retinal artery occlusion; ICD = International Classification of Diseases

© 2023 Hwang DDJ et al. *JAMA Network Open.*

eTable 10. Causes of death among patients with retinal artery occlusion aged <50 years in Korea from 2005 to 2018

Causes of Death (ICD-10 Code)	All		CRAO	Non-central RAO	Age Groups															
					<5		15-19		20-24		25-29		30-34		35-39		40-44		45-49	
	N	%	N	N	N	%	N	%	N	%	N	%	N	%	N	%	N	%	N	%
	**268**	100.0	**120**	**148**	**1**	100.0	**3**	100.0	**4**	100.0	**14**	100.0	**19**	100.0	**31**	100.0	**67**	100.0	**129**	100.0
Neoplasms (C00-D48)	60	22.4	26	34	0	0.0	1	33.3	1	25.0	3	21.4	4	21.1	5	16.1	13	19.4	33	25.6
Diseases of the circulatory system (I00-I99)	59	22.0	28	31	0	0.0	0	0.0	2	50.0	1	7.1	3	15.8	5	16.1	19	28.4	29	22.5
External causes of morbidity (V01-Y89)	41	15.3	18	23	0	0.0	2	66.7	1	25.0	4	28.6	3	15.8	6	19.4	12	17.9	13	10.1
Endocrine, nutritional and metabolic diseases (E00-E88)	31	11.6	16	15	0	0.0	0	0.0	0	0.0	0	0.0	1	5.3	4	12.9	7	10.4	19	14.7
Diseases of the genitourinary system (N00-N98)	21	7.8	10	11	0	0.0	0	0.0	0	0.0	1	7.1	3	15.8	3	9.7	3	4.5	11	8.5
Diseases of the digestive system (K00-K92)	13	4.9	4	9	0	0.0	0	0.0	0	0.0	1	7.1	0	0.0	2	6.5	4	6.0	6	4.7
Symptoms, signs and abnormal clinical and laboratory findings, not elsewhere classified (R00-R99)	13	4.9	4	9	0	0.0	0	0.0	0	0.0	1	7.1	1	5.3	2	6.5	5	7.5	4	3.1
Diseases of the musculoskeletal system and connective tissue (M00-M99)	6	2.2	2	4	0	0.0	0	0.0	0	0.0	1	7.1	1	5.3	2	6.5	0	0.0	2	1.6
Certain infectious and parasitic diseases (A00-B99)	4	1.5	2	2	0	0.0	0	0.0	0	0.0	0	0.0	0	0.0	1	3.2	0	0.0	3	2.3
Mental, Behavioral and Neurodevelopmental disorders (F01-F99)	4	1.5	3	1	0	0.0	0	0.0	0	0.0	0	0.0	1	5.3	1	3.2	0	0.0	2	1.6
Congenital malformations, deformations and chromosomal abnormalities (Q00-Q99)	4	1.5	4	0	1	100.0	0	0.0	0	0.0	0	0.0	1	5.3	0	0.0	2	3.0	0	0.0
Diseases of the nervous system (G00-G98)	3	1.1	1	2	0	0.0	0	0.0	0	0.0	0	0.0	0	0.0	0	0.0	1	1.5	2	1.6
Diseases of the respiratory system (J00-J98,U04)	3	1.1	0	3	0	0.0	0	0.0	0	0.0	1	7.1	0	0.0	0	0.0	0	0.0	2	1.6
Diseases of the skin and subcutaneous tissue (L00-L98)	1	0.4	0	1	0	0.0	0	0.0	0	0.0	0	0.0	0	0.0	0	0.0	0	0.0	1	0.8
Pregnancy, childbirth and the puerperium (O00-O99)	1	0.4	0	1	0	0.0	0	0.0	0	0.0	0	0.0	1	5.3	0	0.0	0	0.0	0	0.0
Diseases of the blood and blood-forming organs and certain disorders involving the immune mechanism (D50-D89)	1	0.4	1	0	0	0.0	0	0.0	0	0.0	1	7.1	0	0.0	0	0.0	0	0.0	0	0.0
Unknown	3	1.1	1	2	0	0.0	0	0.0	0	0.0	0	0.0	0	0.0	0	0.0	1	1.5	2	1.6

RAO = retinal artery occlusion; CRAO = central retinal artery occlusion; ICD = International Classification of Diseases

© 2023 Hwang DDJ et al. *JAMA Network Open.*
