# Supplementary material for: Incidence of Retinal Artery Occlusion and Related Mortality in Korea, 2005 to 2018
Source: JAMA Netw Open. 2023 Mar 10;6(3):e233068. doi: 10.1001/jamanetworkopen.2023.3068 (PMC12527418; doi:10.1001/jamanetworkopen.2023.3068)
Supplement: Supplement 2. — Data Sharing Statement [file jamanetwopen-e233068-s002.pdf]

Data Sharing Statement

Hwang. Incidence of Retinal Artery Occlusion and Related Mortality in Korea, 2005 to 2018. *JAMA Netw Open*. Published March 10, 2023. doi:10.1001/jamanetworkopen.2023.3068

Data
Data available: No

Additional Information
Explanation for why data not available: The datasets used and/or analyzed during the current study are available to researchers who meet the criteria for access to confidential data; requests should be made to the corresponding author upon reasonable request.
